# Characterization of a consensus-designed *trans*-cinnamic acid decarboxylase for styrene biosynthesis

**DOI:** 10.1128/mbio.00714-25

**Published:** 2025-05-23

**Authors:** Ana García-Franco, Jesús de la Torre, Patricia Godoy, Estrella Duque, Carmen López, José A. Gavira, Juan L. Ramos

**Affiliations:** 1 Estación Experimental del Zaidín, Consejo Superior de Investigaciones Científicas, Granada, Spain; 2 Programa de Doctorado en Bioquímica y Biología Molecular, Universidad de Granada 16741, Granada, Spain; 3 Instituto Andaluz de Ciencias de la Tierra, Consejo Superior de Investigaciones Científicas, Armilla, Spain; California Institute of Technology, Pasadena, California, USA; University of Minnesota Twin Cities, St. Paul, Minnesota, USA

**Keywords:** *Pseudomonas*, synthetic biology, styrene biosynthesis, decarboxylases

## Abstract

Microbial biosynthesis of aromatic compounds offers significant advantages over petrochemical methods, which rely on fossil fuels and high energy inputs. Microbial fermentations occur at room temperature and ambient pressure, reducing carbon emissions and energy consumption by up to 90%. Genetic engineering of microbial chassis is key to optimizing biosynthetic processes, enabling efficient production of aromatic compounds from sugars. However, the intrinsic toxicity of these compounds presents challenges. *Pseudomonas putida* DOT-T1E, known for its tolerance to solvents, is ideal for producing toxic compounds. Styrene biosynthesis involves converting phenylalanine into *trans*-cinnamate via phenylalanine ammonia lyase enzymes, followed by decarboxylation to styrene. This second step is challenging, as *trans*-cinnamate decarboxylases have only been described in fungi. PSC1, a consensus protein designed from multiple alignments of fungal ferulate decarboxylases, enables styrene production in *Pseudomonas*. PSC1 is a globular dimer with a molecular mass of 104.7 kDa, high thermal stability (*T*_*m*_ 63°C), and activity at temperatures up to 50°C. The crystal structure of PSC1, determined at 2.1 Å, reveals a homodimer with three domains per monomer. A hydrophobic pocket in domain 2, essential for cofactor and substrate binding, was identified. Mutagenesis shows that Arg175, Glu280, and Glu285 are critical for catalysis, as replacing them with alanine abolished the decarboxylation.

**IMPORTANCE:**

The petrochemical industry is highly polluting due to its use of extremely high temperatures, high pressure, and toxic catalysts. Synthetic biology offers an alternative by enabling the production of many chemicals through cell factories that operate at room temperature and ambient pressure, potentially reducing CO_2_ emissions by up to 90%. We have engineered a solvent-tolerant *Pseudomonas* strain to produce styrene from L-phenylalanine in a two-step process. For the second step, we designed a *de novo* consensus protein that operates efficiently. In this study, we present its physico-chemical properties and unveil its 3D structure.

## INTRODUCTION

The biosynthesis of aromatic compounds from sugars by microbes, such as styrene, styrene oxide, hydroxystyrene, *trans-*cinnamic acid, and others, stands out for its unparalleled efficiency and environmental friendliness (1–9). Unlike chemical processes that heavily rely on fossil fuels and require high pressure, high temperature, and toxic catalyzers, microbial fermentations operate at room temperature and ambient pressure. This not only reduces the carbon footprint but also minimizes energy consumption. Recent studies have highlighted the potential of microbial cell factories in synthesizing valuable chemicals with up to 90% lower CO_2_ emissions compared to their chemical counterparts (10–13).

Genetic engineering plays a crucial role in enabling microbes to efficiently produce aromatic compounds from aromatic amino acids. Bird et al. (14), in their review on the definition of host platforms for the biosynthesis of added-value chemicals, discussed that choosing the right host is the key to optimizing biosynthetic processes and suggested that factors such as the tolerance of the host to substrates and products, understanding the host’s metabolism, and availability of catabolic pathways and tools for stable heterologous gene expression are all essential considerations when designing a successful biosynthetic pathway.

The biosynthesis of aromatic hydrocarbons is challenging because of their intrinsic toxicity to living organisms (15, 16). These compounds disrupt the phospholipid bilayer and interfere with the generation of the proton motive force, collapsing ATP synthesis and eventually leading to cell death (17–19). However, certain strains of the species *Pseudomonas putida* have demonstrated remarkable tolerance to organic solvents, offering a promising solution for the biosynthesis of these toxic compounds. Tolerance to solvents in *P. putida* is achieved through multifactorial defense mechanisms such as efflux pumps that reduce intracellular toxin levels, membrane strengthening via *cis* to *trans* isomerization of unsaturated fatty acids, enhanced synthesis of saturated fatty acids, and activation of general stress responses involving chaperones that fold/refold proteins and enzymes that remove reactive oxygen species (20, 21). The extent of these responses varies with the strain and the toxic compound. The genetic manipulation of *P. putida* benefits from the availability of a robust toolkit that includes the implementation of mini-transposons, expression vectors, regulatable promoters, and wide-host range plasmids (22–24). These tools have paved the way for enhanced control and efficiency in the genetic modifications of microorganisms from this species.

The metabolic pathways of *P. putida* have been extensively studied, revealing intricate networks for balanced growth using different C, N, S, and P sources (17, 25). The chemical synthesis of styrene, known as one of the most polluting petrochemical processes, is being targeted for replacement by eco-friendly biological processes (6, 21). To this end, the highly solvent-tolerant *P. putida* DOT-T1E and S12 are potential hosts for the production of solvents such as styrene (21, 26). Based on the analysis of Udaondo et al. (27), we found that *P. putida* DOT-T1E requires two additional enzymes to convert the aromatic amino acid phenylalanine into styrene, namely: a phenylalanine ammonia lyase (PAL) to convert phenylalanine into *trans-*cinnamic acid and a *trans*-cinnamic acid decarboxylase to convert the latter into styrene (see Fig. S1 at https://doi.org/10.5281/zenodo.15266866). The conversion of phenylalanine into styrene is equimolar, and the concentration of the aromatic amino acid determines the yield of styrene. Under laboratory growth conditions, *P. putida* produces limited amounts of phenylalanine due to dedicated genetic regulation of the biosynthetic genes and appropriate feedback inhibition of the chorismate mutase/prephenate dehydratase (PheA) protein. This protein is involved in the biosynthesis of phenylpyruvate, a precursor of phenylalanine in the biosynthesis of this aromatic amino acid (28, 29). Molina-Santiago et al. (30) engineered DOT-T1E to optimize phenylalanine production, which required inactivating five pathways for phenylalanine degradation and a mutation in the *pheA* gene to remove feedback inhibition by the amino acid. The mutant strain, named CM12-5, produced 340 mg/L of the amino acid. Furthermore, the strain synthesized *trans*-cinnamic acid from phenylalanine using PAL enzymes from cyanobacteria, showing the efficiency of the enzymatic conversion in *P. putida* CM12-5 (21, 30).

Nielsen’s group demonstrated that fungal ferulic acid decarboxylase (FDC EC:4.1.1.102) from *Saccharomyces cerevisiae* can convert *trans*-cinnamic acid into styrene when the gene was cloned and expressed in *Escherichia coli* (6). This reaction is similarly catalyzed by various other fungal ferulic (*trans*-cinnamic) acid decarboxylases (31–35). However, due to the inefficiency of the *Saccharomyces* FDC in *Pseudomonas* (attributed to low gene expression and/or protein instability), a more functional *trans*-cinnamic acid decarboxylase was needed.

In order to achieve efficient styrene production in the solvent-tolerant *P. putida* DOT-T1E, García-Franco et al. (21) employed innovative strategies, including the use of multiple sequence alignment (MSA) to derive consensus proteins as described by Sternke et al. (36). The PSC1 sequence was derived from the MSA of eight fungal FDCs, each exhibiting over 60% identity to the enzyme from *Saccharomyces*. Whilst typical average identities between consensus sequences and MSAs range from 40% to 60% (36, 37), the PSC1, designed with a criterion of >60% identity to *S. cerevisiae* FDC1, shows an identity that ranges from 72% to 80% with the enzymes used for the MSA (see Table S1 at https://doi.org/10.5281/zenodo.15266866).

Fungal ferulic (*trans*-cinnamic) acid decarboxylases, such as those transforming *trans*-cinnamic acid, belong to the UbiD family of decarboxylases. Their biochemical characterization has revealed valuable insights into the role of prenylated FMN (prFMN) as a cofactor (33, 34). Studies by Leys’ lab and others on UbiD decarboxylases from *Aspergillus niger* and *S. cerevisiae* uncovered that the prFMN cofactor exists in two distinct forms: prFMN_iminium_ (active in catalysis) and prFMN_ketimine_ (the inactive form) (see Fig. S2 at https://doi.org/10.5281/zenodo.15266866) (33, 34, 38–41). The biosynthesis of prFMN appears to be a universal process (42, 43) and in *P. putida* is likely to take place as depicted in Fig. S3 at https://doi.org/10.5281/zenodo.15266866, with *in vivo* evidence that in *P. putida* prFMN is incorporated into different apoenzymes, such as PSC1.

To advance the industrial development of styrene biosynthesis in *P. putida* CM12-5, efforts have been directed toward purifying and characterizing the PSC1 enzyme, determining its physico-chemical properties, substrate specificity, and structural characteristics. The high thermostability and substrate specificity of PSC1 make it a promising candidate for enhancing styrene production in microbial systems.

## RESULTS

### Purification of PSC1 protein, molecular mass, and oligomeric state

The *psc*1 gene encodes a 502-amino-acid protein with an estimated molecular mass of 55.4 kDa, which falls within the typical mass range for UbiD family enzymes (55–59 kDa) (44). The PSC1 protein was purified as described in Materials and Methods, and it was kept in solution up to 12–15 mg/mL. SDS-PAGE revealed a series of fractions with a homogeneous protein of approximately 55 kDa (see Fig. S4 at https://doi.org/10.5281/zenodo.15266866), which were pooled for further assays.

To further investigate the size and shape of PSC1, analytical ultracentrifugation sedimentation studies were undertaken. The ratio of the frictional coefficient of PSC1 to that of an ideal globular protein (*f*/*f*_0_ ratio) was found to be 1.258 (see Table S2 at https://doi.org/10.5281/zenodo.15266866), indicating that PSC1 is predominantly globular with some elliptical characteristics. Sedimentation gradient analysis revealed that over 94% of the protein corresponded to a single species with a sedimentation coefficient of 5.19 S (see Fig. S5 at https://doi.org/10.5281/zenodo.15266866). When corrected to standard conditions (the density and viscosity of water at 20°C), the sedimentation coefficient (*S*_20,w_) was calculated to be 5.994 S (see Table S2 at https://doi.org/10.5281/zenodo.15266866). These analyses suggest a molecular mass of 104,691 Da for PSC1, indicating that this enzyme exists as a dimer in solution, consistent with the crystal structure of the *A. niger* and *S. cerevisiae* FDC enzyme.

### Thermal stability

**Fig 1:**
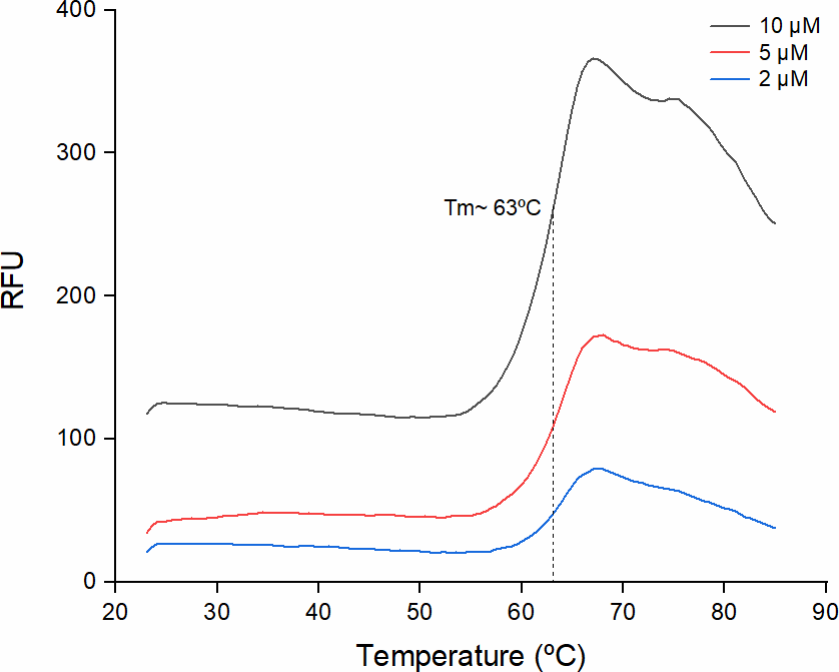
Thermal unfolding behavior of PSC1. The PSC1 protein was purified to homogeneity as described in Materials and Methods. The protein concentration was determined, and the PSC1 protein was diluted in HEPES buffer (pH 7.0) supplemented with 150 mM NaCl to final concentrations of 2, 5, and 10 µM. Then, the effect of the incubation temperature on the thermal stability of PSC1 was assessed by measuring relative fluorescence units (RFUs). The unfolding temperature (*T*_*m*_) was determined using the Bio-Rad iQ5 software.

Following the protocol described in Materials and Methods, the midpoint temperature of the unfolding transition (*T*_*m*_) for PSC1 was found to be 63°C (Fig. 1). The purified protein, stored for 2 weeks at 4°C or 30°C, was stable as the *T*_*m*_ of these samples, yielding an unfolding temperature of 61°C–63°C when tested. This consistency suggests that the PSC1 protein is highly stable, with unfolding temperatures typical of extremophilic proteins. The PSC1 protein entirely unfolded at 80°C, and the fully denatured protein did not refold spontaneously upon incubation for up to 24 h at 4°C or at room temperature.

Nagy et al. (34) reported that the optimal *in vivo* temperature for FDC1 of *S. cerevisiae* is 35°C, but the *T*_*m*_ of FDC1 remains unknown. Given the apparent thermoresistance characteristics of PSC1, we tested *in vivo* the conversion of *trans*-cinnamic acid to styrene over a broad range of temperatures, up to 50°C (higher temperatures could not be tested due to the fast death of cells). We observed rapid conversion of *trans*-cinnamic into styrene at all tested temperatures, with the initial consumption rate being higher at 50°C and 45°C (see Table S3 at https://doi.org/10.5281/zenodo.15266866), i.e., 12.8 ± 0.3 µmol/L·min at 50°C compared to 6.0 ± 0.3 µmol/L·min at 37°C and 4.0 ± 0.3 µmol/L·min at 25°C (see Table S3 at https://doi.org/10.5281/zenodo.15266866). From the data of Nagy et al. (34), we estimated that *in vivo trans*-cinnamic acid decarboxylation by the *Saccharomyces* FDC enzyme in *E. coli* was 4–8 µmol/L·min at 35°C, values similar to those we report here.

### The PSC1 exhibits narrow substrate specificity

Fungal FDC enzymes typically exhibit broad substrate specificity (34), so we tested whether analogs of *trans*-cinnamic acid (see Fig. S6at https://doi.org/10.5281/zenodo.15266866) could serve as potential substrates for PSC1. Our studies with *P. putida* CM12-5 expressing PSC1 revealed that PSC1 effectively decarboxylated *trans*-cinnamic acid into styrene. PSC1 also converted other substrates, albeit at lower rates (Table 1), and in all cases, the corresponding decarboxylation products were detected by headspace gas chromatography-mass spectrometry (GC-MS). Substrates on which PSC1 was active were *p*-coumaric acid, which was converted to 4-vinylphenol (see Fig. S6 at https://doi.org/10.5281/zenodo.15266866), *trans*-ferulic acid, and 3,4-dimethoxycinnamic acid, which yielded 4-vinylguaiacol and 3,4-dimethoxy styrene, respectively. PSC1 failed to decarboxylate chlorogenic acid, caffeic acid, and *trans-*sinapic acid. The inability to decarboxylate these latter substrates is likely due to the presence of bulky substituents, a limitation that has also been reported for the *Saccharomyces* FDC1 enzyme.

**TABLE 1:** Initial consumption rates of *trans-*cinnamic acid and analogs^*a*^

Substrate	Initial consumption rate (µmol/L·min)
*trans-*Cinnamic acid	4.8 ± 0.3
*p-*Coumaric acid	2.0 ± 0.8
Chlorogenic acid	0.3 ± 0.2
*trans*-Ferulic acid	0.9 ± 0.7
3,4-Dimethoxycinnamic acid	0.2 ± 0.1
*trans*-Sinapic acid	0.6 ± 0.4

^*a*^ Resting cells of *Pseudomonas putida* CM12-5 (pPSC1) were prepared as described in Materials and Methods. Substrate concentrations were monitored for 30 min, during which a linear decrease in substrate levels was observed. Data are the mean values and standard deviations from three independent assays.

### PSC1 crystal structure determination and identification of critical catalytic residues

**Fig 2:**
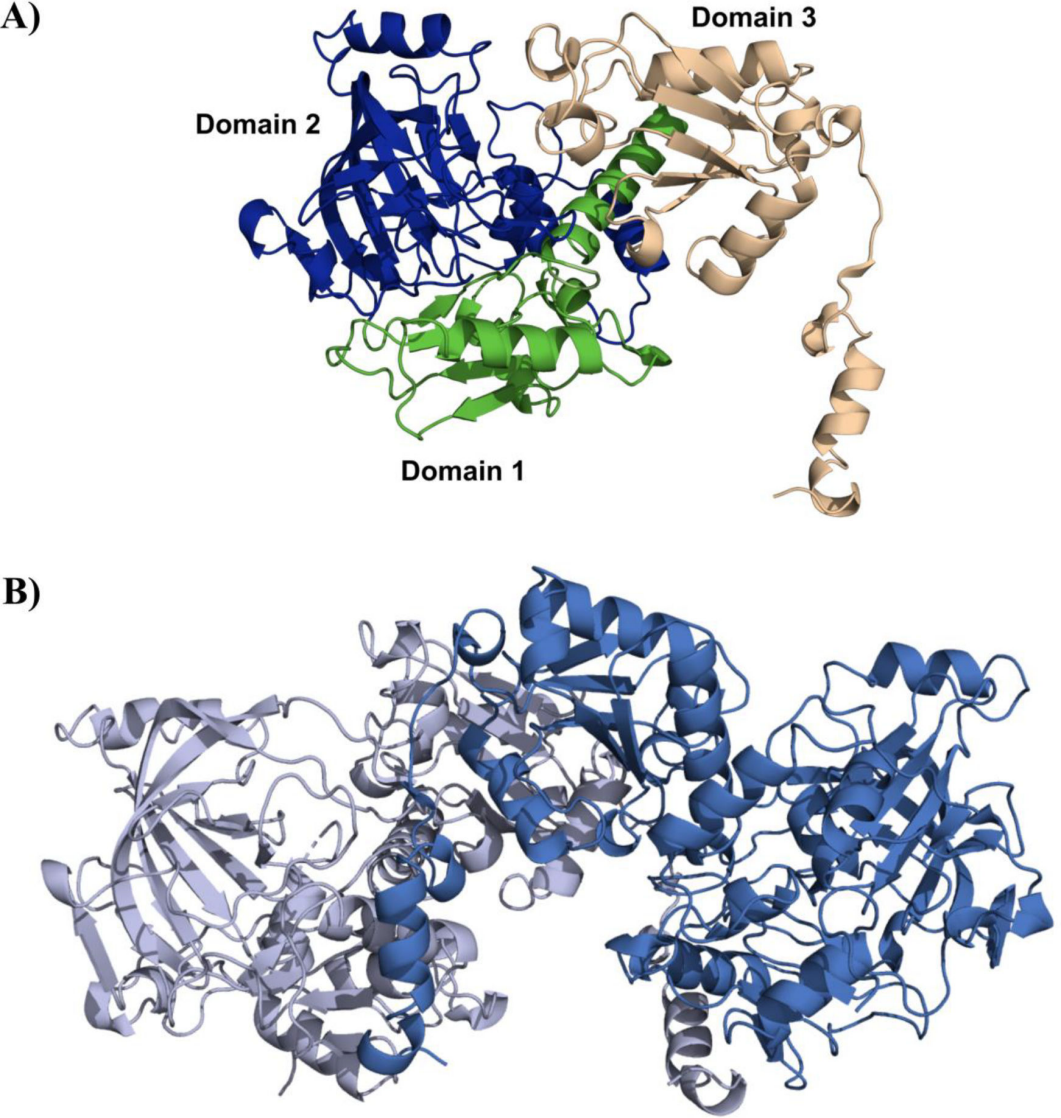
Monomeric and dimeric structure of PSC1. (**A**) Ribbon diagram of the PSC1 monomer, showing the three domains, color-coded as follows: domain 1 (green), domain 2 (blue), and domain 3 (yellow). (**B**) Ribbon diagram of the PSC1 dimer showing each monomer in light and dark blue, showing the role of domain 3 in dimerization.

We aimed to determine the crystal structure of the PSC1 protein. The crystal structure of PSC1 was solved at a resolution of 2.1 Å, belonging to space group P1. Data collection and statistical details are summarized in Table 2. The PSC1 structure is a homodimer, with a Matthews coefficient of 2.15 and 43% solvent content. The dimeric quaternary structure was also confirmed with PISA (45), with an interface buried area of 3,500 Å^2^ and a solvation free energy gain of −58 kcal/mol. The interface is also stabilized by the formation of 3 salt bridges and 29 potential hydrogen bonds. The organization of the secondary structural elements (β-sheets and α-helices) in each monomer resembled that of other deposited 3D structural models of proteins of the UbiD family. Each PSC1 monomer is composed of three domains. Domains 1 and 2 at the N-terminus of the monomer are connected to the third domain by a long alpha-helix (helix 14) (Fig. 2A; see also Fig. S7 at https://doi.org/10.5281/zenodo.15266866). The first domain presents a central four-stranded β-sheet surrounded by six alpha-helices. Helix 4 connects this domain to domain 2, which contains a core β-sheet capped by multiple α-helices (see Fig. S7 at https://doi.org/10.5281/zenodo.15266866). The third domain has a similar structure to that of the second domain, with a core β-sheet that is capped by multiple α-helices, although it is smaller in size. A long, flexible loop connects to the C-terminal helix (α24) and the terminus. PSC1 dimerization takes place through the third domain of each monomer, resulting in U-shape dimer conformation (Fig. 2B), as previously described (31).

**TABLE 2:** Data collection and refinement statistics^*a*^

Parameter	PSC1
Data collection	
Synchrotron/beamline	ESRF/ID30B
Space group	P1
Cell dimensions	
*a*, *b*, *c* (Å)	56.63, 66.28, 72.67
a, b, g (°)	71.67, 73.08, 69.88
Resolution range	52.04–2.10 (2.14–2.10)
Completeness (%)	93.30 (61.90)
Multiplicity	2.2 (1.9)
Mean I/sigma(I)	5.1 (1.0)
Wilson B-factor (Å^2^)	40.07
R-merge	0.124 (1.48)
CC1/2	0.993 (0.619)
Refinement	
No. reflections	50,145 (1,784)
*R*_work_/*R*_free_	21.43/24.73
No. atoms	8,330
Protein	8,010
Ligand/ion	42
Water	278
Root mean square deviations	
Bond lengths (Å)	0.003
Bond angles (°)	0.58
Ramachandran	
Allowed (%)	97.06
Outliers (%)	0.00
Average B-factor (Å^2^)	53.22
Macromolecules	53.39
Ligands	66.55
Solvent	46.50
PDB ID	9GQR

^*a*^ Statistics for the highest-resolution shell are shown in parentheses.

**Fig 3:**
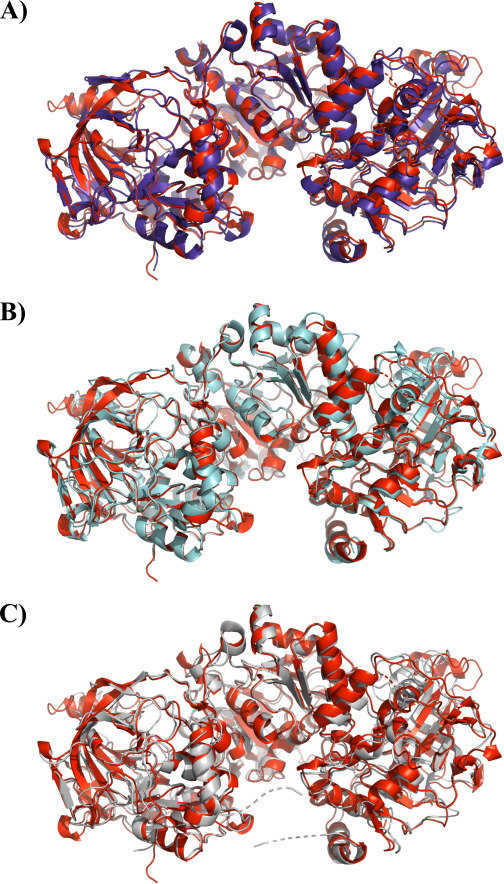
Comparison of the PSC1 dimeric homodimer (red) with (A) FDC1 from *Saccharomyces cerevisiae* (PDB ID 4S13) (purple), (B) FDC1 from *Candida dubliniensis* (PDB ID 4ZAD) (cyan), and (C) FDC1 from *Aspergillus niger* (PDB ID 7NF3) (gray), with RMSD of 0.79, 1.21, and 1.39 Å, respectively.

Structural comparison of PSC1 with entries in the Protein Data Bank (PDB) using PDBeFold (46) revealed a high structural similarity with other crystallized UbiD-family decarboxylases, as expected. The highest similarity was found with the FDC1 from *S. cerevisiae* (79% amino acid sequence identity; *Z*-score = 25.2; and 0.79 Å root mean square deviation [RMSD]; PDB ID 4S13) (Fig. 3A). PSC1 also showed similarity to *Candida dubliniensis* FDC1 (71% amino acid sequence identity; a *Z*-score of 24.2; and a RMSD of 1.21 Å; PDB entry 4ZAD) (Fig. 3B) and the FDC1 from *A. niger* (53% amino acid sequence identity; *Z* = 22.5; and 1.39 Å RMSD; PDB ID 7NF3) (Fig. 3C).

Co-crystallization assays and biochemical studies of FDC from *A. niger* and *S. cerevisiae* revealed that a large hydrophobic pocket, located within domain 2, is involved in interactions with the prFMN cofactor and influences substrate specificity. Alignment of these fungal FDC proteins and PSC1 allowed us to identify that the hydrophobic pocket includes residues I189, Q192, I330, F397, and I398 in PSC1. Using the approach described in Materials and Methods, we generated single amino-acid replacements using site-directed mutagenesis (Table 3). The mutants I189A, Q192A, I330A, F397Y, I398T, and the double mutant F397Y + I398T failed to decarboxylate *trans*-cinnamic acid, suggesting that these mutations disrupted the capacity of the enzyme to bind to its cofactor.

**TABLE 3:** Enzymatic activity of PSC1 mutants compared to the wild type^*a*^

PSC1 mutant	Percentage of activity
PSC1 (control)	100 ± 1.0
I189A	13.8 ± 2.1
Q192A	1.4 ± 0.6
I330A	3.5 ± 1.9
F397Y	4.1 ± 0.8
I398T	0.4 ± 1.2
F397Y + I398T	1.4 ± 2.5
R175A	3.3 ± 0.2
R175K	59.1 ± 1.4
E285A	1.9 ± 5.4
E285D	59.7 ± 0.8

^*a*^ Data are presented as the mean percentage of activity relative to the wild-type PSC1 (set at 100%, equivalent to 4.7 µmol/min) ± standard deviation of three independent assays.

In the *A. niger* FDC protein, the catalytic residues correspond to Arg173, Glu277, and Glu282. Sequence comparison showed that these corresponding residues are invariantly conserved across UbiD family enzymes, with the corresponding residues in PSC1 identified as Arg175, Glu280, and Glu285. This set of residues forms an ionic network within the active site, which is consistent with the decarboxylation mechanism of UbiD enzymes, which involves proton transfer during the reaction (33). As expected, and consistent with previous findings in FDC, replacement of Glu285 with alanine resulted in a mutant devoid of activity (Table 3). Similarly, the replacement of Arg175 with alanine also abolished enzymatic activity. However, when Arg175 was replaced by lysine and Glu285 with aspartate, the resulting mutants retained nearly 60% of the original activity (Table 3).

## DISCUSSION

Decarboxylation is a pivotal biochemical process involving enzymes that utilize a variety of cofactors, such as pyridoxal phosphate, metal ions, and flavin derivatives like FMN and FAD. According to Batyrova et al. (43), over 110 families of decarboxylases exist, each with diverse mechanisms and functions, underscoring their involvement in a broad range of physiological roles (47–50).

The UbiD family of decarboxylases, particularly the FDC enzymes, has raised significant interest due to their ability to convert various substrates into value-added chemicals. For example, McKenna and Nielsen’s group (6) demonstrated the conversion of *trans*-cinnamic acid into styrene using *Escherichia coli* expressing the *S. cerevisiae FDC1* gene. These enzymes are also noteworthy because they use the rare prFMN cofactor (51). UbiD family enzymes can either retain the cofactor, displaying a pale yellow color when purified, or lose it during purification. The solvent-tolerant *P. putida* DOT-T1E can synthesize prFMN (P. Godoy, A. García-Franco, and J. L. Ramos, unpublished data), and our studies suggest that a functional *trans*-cinnamic acid decarboxylase assembles *in vivo*, as confirmed in *P. putida* expressing the *psc1* gene (21). However, the purified PSC1 enzyme was colorless, and absorbance scans indicated that the purified protein corresponded to the apoenzyme. We hypothesize that prFMN binds to the apoenzyme with low affinity or that its binding is unstable. UbiD family proteins that lack prFMN can be reconstructed *in vitro* upon synthesis of the co-factor under anaerobic conditions (32, 34). Furthermore, *in vitro* reconstruction assays with PSC1 are therefore required for comprehensive biochemical characterization.

PSC1 is a consensus-derived protein using the wholesale approach described by Sternke et al. (36). These authors highlighted that consensus sequences derived from MSAs (wholesale approach) represent the evolutionary trajectory of a protein family, often enhancing stability. However, the activity of consensus proteins can increase or decrease relative to extant proteins (36). For PSC1, the wholesale approach proved valuable in designing a protein that works in a prokaryotic microorganism despite its eukaryotic origin (21). Although the wholesale approach does not directly select for activity, conserved sequences often correlate with functional conservation, particularly in active sites and monomer interaction surfaces. This was evident when the structure of the monomer of PSC1 was aligned with that of the *A. niger* FDC1 monomer. Despite only 52% overall sequence conservation, both models superimpose with just 1.39 Å RMSD (see Fig. S7 at https://doi.org/10.5281/zenodo.15266866). Notably, the FDC1 of *A. niger* was not included in the MSA used to derive the PSC1 consensus sequence due to its <60% identity with the *S. cerevisiae* FDC1 enzyme. Consensus proteins derived fseerom both limited and extensive sequence sets have demonstrated enhanced thermotolerance. PSC1 exhibits a *T*_*m*_ of 63°C, consistent with high thermostability. However, a direct comparison with the *T*_*m*_ of extant proteins is not possible due to the lack of available data in the literature. It should be mentioned that a consensus phytase designed from 13 sequences exhibited an increased *T*_*m*_ of 15°C–26°C (52) compared to extant proteins. The high thermostability of PSC1 is further supported by our observation that the rate of conversion of *trans*-cinnamic acid increased with temperature, peaking at 45°C–50°C in *P. putida*, compared to the optimal 35°C for the FDC1 of *S. cerevisiae* expressed in *E. coli* (34).

Several studies have shown that FDC enzymes exhibit broad substrate specificity, decarboxylating *ortho-, meta*-, or *para-*substituted cinnamic acid derivatives. However, despite a good sequence alignment between PSC1 and FDC1 from *A. niger* and *S. cerevisiae* (31, 33, 34, 39, 53), which revealed the conservation of an apolar binding pocket for prFMN cofactor and substrates, PSC1 exhibited a narrower substrate range. PSC1 decarboxylated fewer substrates, including *trans*-cinnamic acid, *p*-coumaric acid, ferulic acid, and 3,4-dimethoxycinnamic acid. Since stability features are likely embedded in the consensus sequence, making the wholesale approach effective for protein stabilization, there may be a trade-off between enhanced stability and substrate recognition. Specifically, increased stability may result in reduced substrate specificity, whereas relaxation of stability constraints may lead to broader substrate recognition. However, this hypothesis remains to be tested.

Sequence comparison and alignment of PSC1 with crystallized proteins identified critical residues in the cofactor/substrate binding region and catalysis. Bailey et al. (33) identified three critical catalytic residues in their *in vitro* assay. Their analysis revealed that residues such as Arg173 and Glu282 in *A. niger* FDC1 are crucial for decarboxylation via proton transfer. Mutants like R173A and E282Q in *A. niger* were found to be catalytically inactive (33, 39) and exhibited altered UV-visible spectra. This was interpreted as a potential ionic network involved in cofactor maturation and catalysis. Our results indicate that the corresponding residues in PSC1, Arg175, and Glu285 are essential for decarboxylation. Notably, lysine can substitute for Arg at position 175, and aspartate can replace Glu at position 285, consistent with the decarboxylation mechanism. This observation is consistent with García-Franco et al. (21), who demonstrated that PSC1 activity is pH dependent, with an optimal pH of 7, underscoring the importance of acidic residues in the decarboxylation process. These findings are in agreement with the results obtained by Bhuiya et al. (33), who reported that FDC1 of *Saccharomyces* exhibited optimal activity at neutral pH.

Nagy et al. (34) used computational simulations to demonstrate that the reaction mechanism in this set of enzymes involves not only the catalytic residues but also the proper co-factor and substrate-binding interactions. These interactions rely on the alignment of the alpha/beta double bond of the substrate with the ribitol tail of prFMN. Their findings suggested that the reaction involves an extended Pi-system associated with the aromatic ring of the substrate, which plays a crucial role in stabilizing the transition state. Their studies also proposed that residues Q192 and I330 contribute to broader substrate specificity (33). The mutation Q192N in FDC1 did not increase the substrate range, while mutants I330V and I330A exhibited modest increases in activity against (E)-3-(2-phenyltiazol-4-yl) acrylic acid. In PSC1, we generated Q192A and I330A mutants, but these mutations led to severe loss of decarboxylation activity. This agrees with our findings that mutations in the cofactor binding residues result in loss of activity, likely due to reduced affinity for prFMN or labile interactions between prFMN and the apoenzyme.

This study highlights the potential of leveraging consensus sequences to design prokaryotic enzymes from eukaryotic organisms that can be stably expressed and function efficiently in prokaryotic systems. Further research into optimizing these designs could advance synthetic biology and industrial biotechnology.

## MATERIALS AND METHODS

### Bacterial strains, plasmids, and culture media

The *E. coli* BL21 (DE3) strain (54) was used for protein overexpression, and *E. coli* DH5α was used for gene cloning (55). *E. coli* cells were grown in LB medium at temperatures ranging from 18°C to 37°C. *P. putida* strains were grown on M9 minimal medium with glucose as the sole C source (56) at 30°C. The plasmids used or constructed in this study are listed in (see Table S4 at https://doi.org/10.5281/zenodo.15266866), while the primers used for mutant generation are listed in (see Table S5 at https://doi.org/10.5281/zenodo.15266866).

### Protein purification

To purify the PSC1 protein, a *Pseudomonas* codon-optimized *psc1* gene was designed and cloned in pET28(b) for expression, with the coding region located downstream of a His-tag sequence. The highest protein yields were obtained when bacteria were grown at 30°C with 1 mM IPTG. For the protein purification, induced cells (1–2 g) were resuspended in 25 mL of lysis buffer (50 mM Tris-HCl, 500 mM NaCl, 1 mM DTT, 10 mM imidazole, and 10% [vol/vol] glycerol) supplemented with an EDTA-free protease inhibitor mixture. Cell lysis was achieved by passing the sample through a French press three times at a pressure of 1,000 psi. The lysate was centrifuged at 6,000 × *g* for 30 min, and the supernatant was filtered using sterile 0.22 µm filters before loading onto a 5 mL His-Trap chelating column (GE Healthcare, St. Gibes, UK). The proteins were eluted using a gradient of 10–500 mM imidazole in the above buffer. Homogeneous protein fractions were dialyzed overnight against a buffer made of 50 mM HEPES (pH 7.0), 150 mM NaCl, and 10% (vol/vol) glycerol. For the sedimentation rate analysis and dynamic light scattering assays, the concentration of glycerol was reduced to 2% (vol/vol) in the dialysis buffer.

### Analytical ultracentrifugation, dynamic light scattering, and differential scanning fluorimetry

An Optima XL-I analytical ultracentrifuge (Beckman-Coulter, Palo Alto, CA, USA) equipped with a UV-visible absorbance detection system was used. Experiments were carried out at 20°C using an AnTi50 rotor, and absorbance measurements were taken at 280 nm. Experimental details and the determination of PSC1 molecular mass are provided at https://doi.org/10.5281/zenodo.15266866. The unfolding temperature (*T*_*m*_) of PSC1 protein was determined as described at https://doi.org/10.5281/zenodo.15266866.

### Plasmid DNA extraction and generation of PSC1 mutant proteins

Highly pure plasmid DNA was obtained using the NZYMiniprep commercial kit (NZYTech) according to the manufacturer’s instructions.

To generate PSC1 mutants, the plasmid pSEVA632_C1, carrying the 1,509 nucleotide sequence encoding the PSC1 protein, was used as a template. Point mutations at positions 175, 189, 192, 285, and 330 were generated by replacing these residues with alanine using the method described by Li and Wilkinson (57). Arg175 was also replaced by lysine and Glu285 by aspartate. Point mutations at positions F397 and I398, as well as a double mutant at these positions, were also generated. They were replaced with tyrosine and threonine, respectively, or both residues were replaced by tyrosine and threonine. The replacements in these two latter positions correspond to the amino acids located in the alignment of PSC1 with *A. niger* FDC1. Plasmids used are listed in (see Table S4 at https://doi.org/10.5281/zenodo.15266866).

### Substrate profile of PSC1

Since we found that the PSC1 protein lost the prFMN cofactor during purification, the substrate profile was assayed *in vivo* according to Nagy et al. (34), except that we used *P. putida* CM12-5 bearing pPSC1, or mutant variants, and the cell suspension was prepared as follows: cultures were allowed to reach a turbidity of ~1 at 660 nm and then the cells were harvested by centrifugation at 6,000 × *g* for 3 min and suspended to reach a cell density of ~10. Cells were incubated at temperatures between 18°C and 50°C and then the substrates were added to reach a final concentration of 0.25 mM. The substrates used in this study included *trans-*cinnamic acid, chlorogenic acid, *p*-coumaric acid, *trans*-ferulic acid, *trans*-sinapic acid, and 3,4-dimethoxycinnamic acid. Their concentrations in the culture medium were monitored over time by HPLC, as described (21), and their decarboxylation products were identified by headspace GC-MS.

### Crystallization of PSC1

PSC1 was freshly purified as described above, concentrated to 10–12 mg/mL and then dialyzed against a buffer made of 50 mM HEPES (pH 7.0), 50 mM NaCl, and 2% (vol/vol) glycerol. Initial crystallization screenings were carried out at 20°C with a dispenser in sitting-drop vapor diffusion plates using commercially available crystallization screens (HR I and II from Hampton Research and Morpheus I and II from Molecular Dimensions). Several conditions produced crystalline material, but the most promising one was grown in condition #6 of HR I containing 200 mM MgCl_2_, 100 mM Tris-HCl (pH 8.5), and 30% (wt/vol) PEG 4K. For data collection, some crystals were soaked with a cryoprotectant solution prepared by adding 15% (vol/vol) of glycerol to the crystallization condition. Crystals with and without cryoprotectant were flash cooled in liquid nitrogen. Diffraction data were collected at the beamline XALOC of the Spanish Synchrotron radiation source, Alba (Barcelona, Spain). Diffraction data were indexed and integrated with XDS (58) and scaled and reduced with AIMLESS (59) of the CCP4 program suite (60). Molecular replacement was done using the best scored AlphaFold2 (61) model as the search model in Molrep (62). Refinement was continued with Refmac (63) and phenix.refine (64) of the CCP4 (60) and PHENIX (65) suites, respectively. Visual inspection, manual building, water positioning, and ligand identification were done in Coot (66). Final refinement was done, including translation-libration-screw parameterization (67), and the quality of the models was regularly verified with MolProbity (68) and the PDB validation server before being deposited in the PDBe (69). Table 2 summarizes crystallographic data statistics and final model characteristics.,

PDBsum server was used to identify the secondary structural elements and define each domain composition (70). The PISA server was used to calculate the most probable quaternary structure assemblies (45). Graphical representation of structural models was prepared with PyMol (71).

## Data Availability

The coordinates and the experimental structure factors have been deposited in the Protein Data Bank with accession code 9GQR.

## References

[1] Kang S-Y, Choi O, Lee JK, Ahn J-O, Ahn JS, Hwang BY, Hong Y-S. 2015. Artificial de novo biosynthesis of hydroxystyrene derivatives in a tyrosine overproducing Escherichia coli strain. Microb Cell Fact 14:78. doi:10.1186/s12934-015-0268-7 26055892 PMC4460750

[2] Lee K, Bang HB, Lee YH, Jeong KJ. 2019. Enhanced production of styrene by engineered Escherichia coli and in situ product recovery (ISPR) with an organic solvent. Microb Cell Fact 18:79. doi:10.1186/s12934-019-1129-6 31053078 PMC6498506

[3] Liang L, Liu R, Foster KEO, Cook S, Cameron JC, Srubar WV, Gill RT. 2020. Genome engineering of E. coli for improved styrene production. Metab Eng 57:74–84. doi:10.1016/j.ymben.2019.09.007 31525473

[4] Liu C, Men X, Chen H, Li M, Ding Z, Chen G, Wang F, Liu H, Wang Q, Zhu Y, Zhang H, Xian M. 2018. A systematic optimization of styrene biosynthesis in Escherichia coli BL21(DE3). Biotechnol Biofuels 11:14. doi:10.1186/s13068-018-1017-z 29416559 PMC5784704

[5] McKenna R, Pugh S, Thompson B, Nielsen DR. 2013. Microbial production of the aromatic building-blocks (S)-styrene oxide and (R)-1,2-phenylethanediol from renewable resources. Biotechnol J 8:1465–1475. doi:10.1002/biot.201300035 23801570

[6] McKenna R, Nielsen DR. 2011. Styrene biosynthesis from glucose by engineered E. coli. Metab Eng 13:544–554. doi:10.1016/j.ymben.2011.06.005 21722749

[7] Nijkamp K, Westerhof RGM, Ballerstedt H, de Bont JAM, Wery J. 2007. Optimization of the solvent-tolerant Pseudomonas putida S12 as host for the production of p-coumarate from glucose. Appl Microbiol Biotechnol 74:617–624. doi:10.1007/s00253-006-0703-0 17111138

[8] Otto M, Wynands B, Marienhagen J, Blank LM, Wierckx N. 2020. Benzoate synthesis from glucose or glycerol using engineered Pseudomonas taiwanensis. Biotechnol J 15:e2000211. doi:10.1002/biot.202000211 32721071

[9] Machas MS, McKenna R, Nielsen DR. 2017. Expanding upon styrene biosynthesis to engineer a novel route to 2-phenylethanol. Biotechnol J 12. doi:10.1002/biot.201700310 28799719

[10] Fackler N, Heijstra BD, Rasor BJ, Brown H, Martin J, Ni Z, Shebek KM, Rosin RR, Simpson SD, Tyo KE, Giannone RJ, Hettich RL, Tschaplinski TJ, Leang C, Brown SD, Jewett MC, Köpke M. 2021. Stepping on the gas to a circular economy: accelerating development of carbon-negative chemical production from gas fermentation. Annu Rev Chem Biomol Eng 12:439–470. doi:10.1146/annurev-chembioeng-120120-021122 33872517

[11] Godar A, Kamoku C, Nielsen D, Wang X. 2021. Synthetic biology strategies to address waste CO2 loss during biofuel production. Curr Opin Environ Sci Health 24:100305. doi:10.1016/j.coesh.2021.100305

[12] Rabinovitch-Deere CA, Oliver JWK, Rodriguez GM, Atsumi S. 2013. Synthetic biology and metabolic engineering approaches to produce biofuels. Chem Rev 113:4611–4632. doi:10.1021/cr300361t 23488968

[13] Ramos JL, Duque E. 2019. Twenty-first-century chemical odyssey: fuels versus commodities and cell factories versus chemical plants. Microb Biotechnol 12:200–209. doi:10.1111/1751-7915.13379 30793487 PMC6389845

[14] Bird LJ, Mickol RL, Eddie BJ, Thakur M, Yates MD, Glaven SM. 2023. Marinobacter: a case study in bioelectrochemical chassis evaluation. Microb Biotechnol 16:494–506. doi:10.1111/1751-7915.14170 36464922 PMC9948230

[15] Heipieper HJ, Martínez PM. 2018. Toxicity of hydrocarbons to microorganisms, p 335–344. In Krell T (ed), Cellular ecophysiology of microbe: hydrocarbon and lipid interactions. Springer, Cham.

[16] Sikkema J, de Bont JA, Poolman B. 1995. Mechanisms of membrane toxicity of hydrocarbons. Microbiol Rev 59:201–222. doi:10.1128/mr.59.2.201-222.1995 7603409 PMC239360

[17] Udaondo Z, Molina L, Daniels C, Gómez MJ, Molina-Henares MA, Matilla MA, Roca A, Fernández M, Duque E, Segura A, Ramos JL. 2013. Metabolic potential of the organic-solvent tolerant Pseudomonas putida DOT-T1E deduced from its annotated genome. Microb Biotechnol 6:598–611. doi:10.1111/1751-7915.12061 23815283 PMC3918161

[18] Ramos J-L, Sol Cuenca M, Molina-Santiago C, Segura A, Duque E, Gómez-García MR, Udaondo Z, Roca A. 2015. Mechanisms of solvent resistance mediated by interplay of cellular factors in Pseudomonas putida. FEMS Microbiol Rev 39:555–566. doi:10.1093/femsre/fuv006 25934123

[19] Bitzenhofer NL, Höfel C, Thies S, Weiler AJ, Eberlein C, Heipieper HJ, Batra-Safferling R, Sundermeyer P, Heidler T, Sachse C, Busche T, Kalinowski J, Belthle T, Drepper T, Jaeger K-E, Loeschcke A. 2024. Exploring engineered vesiculation by Pseudomonas putida KT2440 for natural product biosynthesis. Microb Biotechnol 17:e14312. doi:10.1111/1751-7915.14312 37435812 PMC10832525

[20] García-Franco A, Godoy P, Duque E, Ramos JL. 2023. Insights into the susceptibility of Pseudomonas putida to industrially relevant aromatic hydrocarbons that it can synthesize from sugars. Microb Cell Fact 22:22. doi:10.1186/s12934-023-02028-y 36732770 PMC9893694

[21] García-Franco A, Godoy P, Duque E, Ramos JL. 2024. Engineering styrene biosynthesis: designing a functional trans-cinnamic acid decarboxylase in Pseudomonas. Microb Cell Fact 23:69. doi:10.1186/s12934-024-02341-0 38419048 PMC10903017

[22] Arce-Rodríguez A, Benedetti I, Borrero-de Acuña JM, Silva-Rocha R, de Lorenzo V. 2023. Standardization of inducer-activated broad host range expression modules: debugging and refactoring an alkane-responsive AlkS/ PalkB device. Synth Biol 6:1–11. doi:10.1093/synbio/ysab030

[23] Martínez-García E, Nikel PI, Aparicio T, de Lorenzo V. 2014. Pseudomonas 2.0: genetic upgrading of P. putida KT2440 as an enhanced host for heterologous gene expression. Microb Cell Fact 13:159. doi:10.1186/s12934-014-0159-3 25384394 PMC4230525

[24] Martínez-García E, de Lorenzo V. 2017. Molecular tools and emerging strategies for deep genetic/genomic refactoring of Pseudomonas. Curr Opin Biotechnol 47:120–132. doi:10.1016/j.copbio.2017.06.013 28738232

[25] Nogales J, Mueller J, Gudmundsson S, Canalejo FJ, Duque E, Monk J, Feist AM, Ramos JL, Niu W, Palsson BO. 2020. High-quality genome-scale metabolic modelling of Pseudomonas putida highlights its broad metabolic capabilities. Environ Microbiol 22:255–269. doi:10.1111/1462-2920.14843 31657101 PMC7078882

[26] Webb JP, Paiva AC, Rossoni L, Alstrom-Moore A, Springthorpe V, Vaud S, Yeh V, Minde D-P, Langer S, Walker H, Hounslow A, Nielsen DR, Larson T, Lilley K, Stephens G, Thomas GH, Bonev BB, Kelly DJ, Conradie A, Green J. 2022. Multi-omic based production strain improvement (MOBpsi) for bio-manufacturing of toxic chemicals. Metab Eng 72:133–149. doi:10.1016/j.ymben.2022.03.004 35289291

[27] Udaondo Z, Molina L, Segura A, Duque E, Ramos JL. 2016. Analysis of the core genome and pangenome of Pseudomonas putida. Environ Microbiol 18:3268–3283. doi:10.1111/1462-2920.13015 26261031

[28] Loeschcke A, Thies S. 2020. Engineering of natural product biosynthesis in Pseudomonas putida. Curr Opin Biotechnol 65:213–224. doi:10.1016/j.copbio.2020.03.007 32498036

[29] Molina-Henares MA, García-Salamanca A, Molina-Henares AJ, de la Torre J, Herrera MC, Ramos JL, Duque E. 2009. Functional analysis of aromatic biosynthetic pathways in Pseudomonas putida KT2440. Microb Biotechnol 2:91–100. doi:10.1111/j.1751-7915.2008.00062.x 21261884 PMC3815424

[30] Molina-Santiago C, Cordero BF, Daddaoua A, Udaondo Z, Manzano J, Valdivia M, Segura A, Ramos J-L, Duque E. 2016. Pseudomonas putida as a platform for the synthesis of aromatic compounds. Microbiology (Reading) 162:1535–1543. doi:10.1099/mic.0.000333 27417954

[31] Bhuiya MW, Lee SG, Jez JM, Yu O. 2015. Structure and mechanism of ferulic acid decarboxylase (FDC1) from Saccharomyces cerevisiae. Appl Environ Microbiol 81:4216–4223. doi:10.1128/AEM.00762-15 25862228 PMC4524143

[32] Richard P, Viljanen K, Penttilä M. 2015. Overexpression of PAD1 and FDC1 results in significant cinnamic acid decarboxylase activity in Saccharomyces cerevisiae. AMB Express 5:12. doi:10.1186/s13568-015-0103-x 25852989 PMC4384992

[33] Bailey SS, Payne KAP, Fisher K, Marshall SA, Cliff MJ, Spiess R, Parker DA, Rigby SEJ, Leys D. 2018. The role of conserved residues in Fdc decarboxylase in prenylated flavin mononucleotide oxidative maturation, cofactor isomerization, and catalysis. J Biol Chem 293:2272–2287. doi:10.1074/jbc.RA117.000881 29259125 PMC5818171

[34] Nagy EZA, Nagy CL, Filip A, Nagy K, Gál E, Tőtős R, Poppe L, Paizs C, Bencze LC. 2019. Exploring the substrate scope of ferulic acid decarboxylase (FDC1) from Saccharomyces cerevisiae. Sci Rep 9. doi:10.1038/s41598-018-36977-x PMC6345843 30679592

[35] Duță H, Filip A, Nagy LC, Nagy EZA, Tőtős R, Bencze LC. 2022. Toolbox for the structure-guided evolution of ferulic acid decarboxylase (FDC). Sci Rep 12:3347. doi:10.1038/s41598-022-07110-w 35232989 PMC8888657

[36] Sternke M, Tripp KW, Barrick D. 2019. Consensus sequence design as a general strategy to create hyperstable, biologically active proteins. Proc Natl Acad Sci USA 116:11275–11284. doi:10.1073/pnas.1816707116 31110018 PMC6561275

[37] Sternke M, Tripp KW, Barrick D. 2020. The use of consensus sequence information to engineer stability and activity in proteins. Methods Enzymol 643:149–179. doi:10.1016/bs.mie.2020.06.001 32896279 PMC8098710

[38] Ferguson KL, Arunrattanamook N, Marsh ENG. 2016. Mechanism of the novel prenylated flavin-containing enzyme ferulic acid decarboxylase probed by isotope effects and linear free-energy relationships. Biochemistry 55:2857–2863. doi:10.1021/acs.biochem.6b00170 27119435

[39] Payne KAP, White MD, Fisher K, Khara B, Bailey SS, Parker D, Rattray NJW, Trivedi DK, Goodacre R, Beveridge R, Barran P, Rigby SEJ, Scrutton NS, Hay S, Leys D. 2015. New cofactor supports α,β-unsaturated acid decarboxylation via 1,3-dipolar cycloaddition. Nature 522:497–501. doi:10.1038/nature14560 26083754 PMC4988494

[40] Roberts GW, Leys D. 2022. Structural insights into UbiD reversible decarboxylation. Curr Opin Struct Biol 75:102432. doi:10.1016/j.sbi.2022.102432 35843126

[41] Bloor S, Michurin I, Titchiner GR, Leys D. 2023. Prenylated flavins: structures and mechanisms. FEBS J 290:2232–2245. doi:10.1111/febs.16371 35073609

[42] Wang P-H, Khusnutdinova AN, Luo F, Xiao J, Nemr K, Flick R, Brown G, Mahadevan R, Edwards EA, Yakunin AF. 2018. Biosynthesis and activity of prenylated FMN cofactors. Cell Chem Biol 25:560–570. doi:10.1016/j.chembiol.2018.02.007 29551348

[43] Batyrova KA, et al.. 2020. Biocatalytic in vitro and in vivo FMN prenylation and (de)carboxylase activation. ACS Chem Biol 15:1874–1882. 32579338 10.1021/acschembio.0c00136

[44] Jacewicz A, Izumi A, Brunner K, Schnell R, Schneider G. 2013. Structural insights into the UbiD protein family from the crystal structure of PA0254 from Pseudomonas aeruginosa. PLoS One 8:e63161. doi:10.1371/journal.pone.0063161 23671667 PMC3650080

[45] Krissinel E, Henrick K. 2007. Inference of macromolecular assemblies from crystalline state. J Mol Biol 372:774–797. doi:10.1016/j.jmb.2007.05.022 17681537

[46] Krissinel E, Henrick K. 2004. Secondary-structure matching (SSM), a new tool for fast protein structure alignment in three dimensions. Acta Crystallogr D Biol Crystallogr 60:2256–2268. doi:10.1107/S0907444904026460 15572779

[47] Vladimirova A, Patskovsky Y, Fedorov AA, Bonanno JB, Fedorov EV, Toro R, Hillerich B, Seidel RD, Richards NGJ, Almo SC, Raushel FM. 2016. Substrate distortion and the catalytic reaction mechanism of 5-carboxyvanillate decarboxylase. J Am Chem Soc 138:826–836. doi:10.1021/jacs.5b08251 26714575 PMC4732527

[48] Li T, Huo L, Pulley C, Liu A. 2012. Decarboxylation mechanisms in biological system. Bioorg Chem 43:2–14. doi:10.1016/j.bioorg.2012.03.001 22534166

[49] Anand R, Dorrestein PC, Kinsland C, Begley TP, Ealick SE. 2002. Structure of oxalate decarboxylase from Bacillus subtilis at 1.75 A resolution. Biochemistry 41:7659–7669. doi:10.1021/bi0200965 12056897

[50] Moomaw EW, Angerhofer A, Moussatche P, Ozarowski A, García-Rubio I, Richards NGJ. 2009. Metal dependence of oxalate decarboxylase activity. Biochemistry 48:6116–6125. doi:10.1021/bi801856k 19473032 PMC2801813

[51] Marshall SA, Fisher K, Ní Cheallaigh A, White MD, Payne KAP, Parker DA, Rigby SEJ, Leys D. 2017. Oxidative maturation and structural characterization of prenylated FMN binding by UbiD, a decarboxylase involved in bacterial ubiquinone biosynthesis. J Biol Chem 292:4623–4637. doi:10.1074/jbc.M116.762732 28057757 PMC5377778

[52] Lehmann M, Loch C, Middendorf A, Studer D, Lassen SF, Pasamontes L, van Loon APGM, Wyss M. 2002. The consensus concept for thermostability engineering of proteins: further proof of concept. Protein Eng 15:403–411. doi:10.1093/protein/15.5.403 12034860

[53] Mukai N, Masaki K, Fujii T, Kawamukai M, Iefuji H. 2010. PAD1 and FDC1 are essential for the decarboxylation of phenylacrylic acids in Saccharomyces cerevisiae. J Biosci Bioeng 109:564–569. doi:10.1016/j.jbiosc.2009.11.011 20471595

[54] Studier FW, Moffatt BA. 1986. Use of bacteriophage T7 RNA polymerase to direct selective high-level expression of cloned genes. J Mol Biol 189:113–130. doi:10.1016/0022-2836(86)90385-2 3537305

[55] Grant SG, Jessee J, Bloom FR, Hanahan D. 1990. Differential plasmid rescue from transgenic mouse DNAs into Escherichia coli methylation-restriction mutants. Proc Natl Acad Sci USA 87:4645–4649. doi:10.1073/pnas.87.12.4645 2162051 PMC54173

[56] Abril MA, Michan C, Timmis KN, Ramos JL. 1989. Regulator and enzyme specificities of the TOL plasmid-encoded upper pathway for degradation of aromatic hydrocarbons and expansion of the substrate range of the pathway. J Bacteriol 171:6782–6790. doi:10.1128/jb.171.12.6782-6790.1989 2687253 PMC210577

[57] Li S, Wilkinson MF. 1997. Site-directed mutagenesis: a two-step method using PCR and DpnI. Biotechniques 23:588–590. doi:10.2144/97234bm05 9343667

[58] Kabsch W. 2010. XDS. Acta Crystallogr D Biol Crystallogr 66:125–132. doi:10.1107/S0907444909047337 20124692 PMC2815665

[59] Evans PR, Murshudov GN. 2013. How good are my data and what is the resolution? Acta Crystallogr D Biol Crystallogr 69:1204–1214. doi:10.1107/S0907444913000061 23793146 PMC3689523

[60] N. 4 Collaborative Computational Project. 1994. The CCP4 suite: programs for protein crystallography. Acta Crystallogr D Biol Crystallogr 50:760–763. doi:10.1107/S0907444994003112 15299374

[61] Jumper J, Evans R, Pritzel A, Green T, Figurnov M, Ronneberger O, Tunyasuvunakool K, Bates R, Žídek A, Potapenko A, et al.. 2021. Highly accurate protein structure prediction with AlphaFold. Nature 596:583–589. doi:10.1038/s41586-021-03819-2 34265844 PMC8371605

[62] Vagin A, Teplyakov A. 2010. Molecular replacement with MOLREP. Acta Crystallogr D Biol Crystallogr 66:22–25. doi:10.1107/S0907444909042589 20057045

[63] Murshudov GN, Skubák P, Lebedev AA, Pannu NS, Steiner RA, Nicholls RA, Winn MD, Long F, Vagin AA. 2011. REFMAC5 for the refinement of macromolecular crystal structures. Acta Crystallogr D Biol Crystallogr 67:355–367. doi:10.1107/S0907444911001314 21460454 PMC3069751

[64] Afonine PV, Mustyakimov M, Grosse-Kunstleve RW, Moriarty NW, Langan P, Adams PD. 2010. Joint X-ray and neutron refinement with phenix.refine. Acta Crystallogr D Biol Crystallogr 66:1153–1163. doi:10.1107/S0907444910026582 21041930 PMC2967420

[65] Adams PD, Afonine PV, Bunkóczi G, Chen VB, Davis IW, Echols N, Headd JJ, Hung L-W, Kapral GJ, Grosse-Kunstleve RW, McCoy AJ, Moriarty NW, Oeffner R, Read RJ, Richardson DC, Richardson JS, Terwilliger TC, Zwart PH. 2010. PHENIX: a comprehensive Python-based system for macromolecular structure solution. Acta Crystallogr D Biol Crystallogr 66:213–221. doi:10.1107/S0907444909052925 20124702 PMC2815670

[66] Emsley P, Lohkamp B, Scott WG, Cowtan K. 2010. Features and development of Coot. Acta Crystallogr D Biol Crystallogr 66:486–501. doi:10.1107/S0907444910007493 20383002 PMC2852313

[67] Painter J, Merritt EA. 2006. TLSMD web server for the generation of multi-group TLS models . J Appl Crystallogr 39:109–111. doi:10.1107/S0021889805038987

[68] Chen VB, Arendall WB 3rd, Headd JJ, Keedy DA, Immormino RM, Kapral GJ, Murray LW, Richardson JS, Richardson DC. 2010. MolProbity: all-atom structure validation for macromolecular crystallography. Acta Crystallogr D Biol Crystallogr 66:12–21. doi:10.1107/S0907444909042073 20057044 PMC2803126

[69] Velankar S, Best C, Beuth B, Boutselakis CH, Cobley N, Sousa Da Silva AW, Dimitropoulos D, Golovin A, Hirshberg M, John M, Krissinel EB, Newman R, Oldfield T, Pajon A, Penkett CJ, Pineda-Castillo J, Sahni G, Sen S, Slowley R, Suarez-Uruena A, Swaminathan J, van Ginkel G, Vranken WF, Henrick K, Kleywegt GJ. 2010. PDBe: protein data bank in Europe. Nucleic Acids Res 38:D308–17. doi:10.1093/nar/gkp916 19858099 PMC2808887

[70] Laskowski RA, Jabłońska J, Pravda L, Vařeková RS, Thornton JM. 2018. PDBsum: structural summaries of PDB entries. Protein Sci 27:129–134. doi:10.1002/pro.3289 28875543 PMC5734310

[71] DeLano WL. 2002. Pymol: an open-source molecular graphics tool. CCP4 Newsletter on Protein Crystallography

